# Protozoa Drive the Dynamics of Culturable Biocontrol Bacterial Communities

**DOI:** 10.1371/journal.pone.0066200

**Published:** 2013-06-26

**Authors:** Maren Stella Müller, Stefan Scheu, Alexandre Jousset

**Affiliations:** Georg August University Göttingen, J. F. Blumenbach Institute of Zoology and Anthropology, Göttingen, Germany; Wageningen University, The Netherlands

## Abstract

Some soil bacteria protect plants against soil-borne diseases by producing toxic secondary metabolites. Such beneficial biocontrol bacteria can be used in agricultural systems as alternative to agrochemicals. The broad spectrum toxins responsible for plant protection also inhibit predation by protozoa and nematodes, the main consumers of bacteria in soil. Therefore, predation pressure may favour biocontrol bacteria and contribute to plant health. We analyzed the effect of *Acanthamoeba castellanii* on semi-natural soil bacterial communities in a microcosm experiment. We determined the frequency of culturable bacteria carrying genes responsible for the production of the antifungal compounds 2,4-diacetylphloroglucinol (DAPG), pyrrolnitrin (PRN) and hydrogen cyanide (HCN) in presence and absence of *A. castellanii*. We then measured if amoebae affected soil suppressiveness in a bioassay with sugar beet seedlings confronted to the fungal pathogen *Rhizoctonia solani*. Amoebae increased the frequency of both DAPG and HCN positive bacteria in later plant growth phases (2 and 3 weeks), as well as the average number of biocontrol genes per bacterium. The abundance of DAPG positive bacteria correlated with disease suppression, suggesting that their promotion by amoebae may enhance soil health. However, the net effect of amoebae on soil suppressiveness was neutral to slightly negative, possibly because amoebae slow down the establishment of biocontrol bacteria on the recently emerged seedlings used in the assay. The results indicate that microfaunal predators foster biocontrol bacterial communities. Understanding interactions between biocontrol bacteria and their predators may thus help developing environmentally friendly management practices of agricultural systems.

## Introduction

Various soil-dwelling bacteria improve plant health by inhibiting pathogens, and have been increasingly investigated during the last decades as biocontrol agents to replace pesticides in agriculture. Biocontrol bacteria produce antifungal secondary metabolites suppressing pathogens and have been shown to reduce disease severity in agricultural systems [1] and contribute to the high productivity of species-rich grasslands [2]. In order to efficiently use beneficial bacteria to increase plant yield, there is the need to better understand the factors driving their fitness in soils. Numerous strains with a high biocontrol potential have been isolated and tested *in vitro*, but their commercial application is often limited by their low persistence in soil in the field [3]. To survive in soil and in the rhizosphere, bacteria must compete with the indigenous microflora and resist predation [4]. Especially predation by protozoa is a major selection pressure that shapes the structure of bacterial communities in the soil and the rhizosphere [5], [6], as well as the competitiveness of single strains [7]. Many bacterial secondary metabolites known for their activity against soil pathogens are also active against protozoa [8], [9], [10]. In laboratory systems, biocontrol bacteria producing secondary metabolites outcompete non-toxic ones when confronted with protozoa [4], [7], [11]. Due to this protective effect, we expected predation by protozoa in soils to promote bacteria producing biocontrol secondary metabolites by preferentially consuming non-producer bacteria, thereby improving the potential of soil bacterial communities to inhibit plant pathogens.

We investigated the impact of predation at the community level, with a particular focus on fluorescent pseudomonads, one of the most intensively studied groups of biocontrol bacteria [1]. Pseudomonads produce a wealth of antimicrobial secondary metabolites and extracellular enzymes that inhibit other bacteria, fungi, protozoa and nematodes [12]. Secondary metabolites include 2,4-diacetylphloroglucinol (DAPG) and pyrrolnitrin (PRN), two broad spectrum antifungal compounds, and hydrogen cyanide (HCN), an inhibitor of the respiratory electron transport system [13]. These compounds efficiently suppress phytopathogenic fungi [12], and also efficiently protect bacteria against nematodes and protozoa [9], [14], [15]. We tested if predation by protozoa increases the numbers of bacteria carrying genes responsible for the synthesis of these toxins and if this results in improved soil suppressiveness against phytopathogens.

We set up microcosms with barley seedlings growing in soil containing semi-natural bacterial communities and the model protozoan predator *Acanthamoeba castellanii.* We measured the abundance of total pseudomonads and the frequency of bacteria carrying the functional genes responsible for the synthesis of DAPG, PRN and HCN. Further, we tested if predation induced functional shifts in bacterial communities resulting in increased plant protection against pathogens in a biocontrol assay with sugar beet and *Rhizoctonia solani*.

## Materials and Methods

### Preparation of protozoa-free bacterial communities

In order to measure the effect of protozoa on soil microbial communities, we first established protozoa-free, semi-natural bacterial communities that were submitted to predation by *A. castellanii*. Soil from the Jena Experiment field site, Germany (Eutric luvisol; for details see Roscher *et al.* [16]), was dried at room temperature for 24 h and one volume of soil was suspended in three volumes of distilled water of 4°C. Bacteria were extracted on ice according to Priemé *et al.* [17] with few modifications: The soil slurry was ultrasonicated for 10 s to detach bacteria from soil particles. The suspension was shaken (300 rpm) for 30 to 60 min and centrifuged at 150 g for 5 min at 4°C to remove soil debris. The supernatant was filtered through cotton discs for manual milk filtration (160 mm), and centrifuged on Percoll (GE Healthcare Bio-Sciences AB, Uppsala, Sweden; density 1.13 g mL^−1^). The upper phase containing bacteria was then filtered through 5 and 1.2 µm membranes to remove protozoa [5]. Filters were changed at regular intervals to avoid contamination by small protozoa (flagellates). The filtrate with bacteria was decanted in tissue culture flasks and incubated at 14°C [6]. After one week the bacterial suspension was checked for contamination by flagellates under an inverted microscope. Prior to use the biofilm in the tissue culture flasks was resuspended in an ultrasonic bath (Bandelin electronic, Berlin, Germany) (10% power, 25 s) at 20°C, centrifuged (4600 rpm, 15 min) and washed twice in Page Amoeba Saline (PAS) [18].

### Amoebae

Amoebae (*A. castellanii*) were isolated from a woodland soil [19] and grown axenically on PGY medium (peptone 10 g L^−1^, glucose 10 g L^−1^, yeast extract 5 g L^−1^) in tissue culture flasks at 14°C [6]. Prior to the experiment, cultures were washed twice by gentle centrifugation (300× g, 30 s). The pellet was resuspended in PAS and stored at 14°C. Cell concentration was determined with a Neubauer counting chamber.

### Plants

Barley (*Hordeum vulgare*) seeds were surface-sterilised as described elsewhere [20]. Briefly, seeds were dehusked in 50% sulphuric acid for 70 min, washed two times with H_2_O for 5 min and once with 1% NaHCO_3_ to neutralise acidity. Dehusked seeds were surface sterilized with 2% AgNO_3_ for 20 min, washed twice for 5 min with 1% NaCl and H_2_O, four times with H_2_O and germinated on 1.5% water agar at 24°C in the dark.

### Microcosm setup

Soil from the Jena experiment field site (see above) was sieved through 2 mm mesh to remove plant debris, macrofauna and stones. Microcosms consisted of 20×300 mm glass tubes filled with 40 g of a 1∶1 mix of soil and quartz sand (grain size 0.1–0.5 mm). Tubes were closed with a cotton plug and aluminium foil, and autoclaved (121°C, 20 min).

Sterile microcosms were inoculated with 2 mL of a protozoa free soil bacteria suspension (4*10^6^ bacteria mL^−1^) and soil moisture was adjusted with sterile water to ensure that the soil was moist but not wet. Microcosms were incubated for one week at 20°C to permit bacterial growth. Barley seedlings were transferred into microcosms inoculated with soil bacterial suspension. After four days microcosms were inoculated with 1 mL amoebae suspension (2*10^5^ amoebae mL^−1^), or 1 mL PAS as control. Eight microcosms were set up for each treatment and sampling date. Plants were grown at 21°C with 12 h of light (250 µmol s^−1^ m^−2^).

### Enumeration of soil bacteria and protozoa

Microcosms were destructively sampled 0, 7, 14 and 21 days after inoculation with protozoa. For each sampling date and treatment, eight microcosms were harvested. Barley roots were shaken in 10 mL 0.1× phosphate buffer saline (PBS) for 1 h to extract rhizosphere bacteria, dried (50°C, 24 h) and weighed. Total aerobic bacteria and fluorescent pseudomonads were enumerated by serial dilution plating on TSA (tryptic soy broth 3 g L^−1^, agar 15 g L^−1^) and Gould's S1 agar plates [21], respectively. Colonies were enumerated after incubation at 24°C for at least 48 h and density expressed as CFUs per plant. Amoebae were enumerated by Most Probable Number (MPN) using *Pseudomonas fluorescens* CHA19 as food source as described elsewhere [11].

### Colony PCR

For each sample, nine colonies were picked from the Gould's S1 plates, heat lysed [22], and the presence of the biocontrol genes *phlD*, *hcnAB* and *prnD* were assessed by PCR as previously described [23], [24], [25]. PCR was carried out in 20 µL reaction mixtures containing 1× KAPA2G Buffer B, 1× KAPA Enhancer 1, 0.2 mM dNTP mix, 0.325 µM of each primer and 0.5 U of KAPA2G Robust DNA Polymerase (PEQLAB, Erlangen, Germany). Amplifications were performed with the following cycling program: initial denaturation at 95°C for 10 min, followed by 40 cycles of denaturation at 95°C for 30 s, primer annealing at 67°C (*phlD*, *hcnAB*) or 68°C (*prnD*) for 45 s and extension at 72°C for 1 min, and a 10 min final extension step at 72°C. The presence of the corresponding amplicons was assessed on 1.5% agarose gel stained with ethidium bromide.

### Biocontrol assay on soil suppressiveness against*Rhizoctonia solani*

The influence of amoebae on the antagonistic potential of the bacterial community against phytopathogens was tested in a biocontrol assay including sugar beet seeds (*Beta vulgaris* L. cv. Belinda) and the pathogen *Rhizoctonia solani* Kühn (AG 2–2 IIIB) as described in Latz *et al.* [2]. The sugar beet - *Rhizoctonia* combination allows efficient screening of pathogen development. Especially the Belinda cultivar is sensitive to a broad range of pathogens and is used as bait plant to isolate generalist soil-borne pathogens; it rapidly develops infection symptoms and dies in absence of protective bacteria [2]. The use of this plant-pathogen system is an extension of a bait plant system: a plant vulnerable to infection by various pathogens may allow estimating pathogen virulence and as a reverse function soil suppressiveness, while avoiding potential biases due to plant-driven accumulation of protective bacteria that might only be able to protect the host plant while being of marginal relevance for other plant species [2]. Briefly, 40 g root free soil from the last harvest at day 21 (8 microcosms without amoebae, 8 microcosms with amoebae) were transfered into autoclaved Magenta boxes (7.4×7.4×9.7 cm) and rewetted with 500 µL sterile H_2_O. Eight sugar beet seeds (99.9% germination rate) were added to each box below the soil surface. Subsequently, half of a barley seed infested with *R. solani* was placed in the center of the box. The boxes were incubated at 21°C with 12 h light (250 µmol s^−1^ m^−2^). Infection was characterized by counting brown roots, stems, leaves and snapped stems of the sugar beet germ buds over a period of 19 days.

### Statistical analyses

The effect of amoebae and time on the frequency of *phlD*, *hcnAB* and *prnD* positive isolates was analyzed with a Poisson GLM. The effect of amoebae as well as the abundance of biocontrol genes on disease development (brown roots, stems, leaves and snapped stems of sugar beet seedlings by *R. solani*) was assessed with a random intercept mixed effect model with Poisson distribution investigating the effect of the amoebae and the abundance of bacteria carrying *phlD*, *hcnAB* or *prnD* genes (as measured at the end of the first experiment) on disease development over time. Each symptom was analyzed separately. All analyses were carried out with R 2.12 (R core development Team, Vienna, Austria).

## Results

### Barley root growth

In this microcosm assay we investigated whether predation by amoebae affects biocontrol bacterial communities associated with the production of antifungal secondary metabolites. Barley roots were weighed to examine the influence of amoebae on root growth. The fresh weight (F_3,54_ = 46.1, P = <0.001) and the dry weight (F_3,54_ = 4.3, P = 0.009) significantly increased with time. Amoebae did not affect barley root growth (F_1,54_ = 2.6, P = 0.114 and F_1,54_ = 0.3, P = 0.60 for fresh weight and dry weight, respectively).

### Dynamics of bacterial and protozoan density

The total number of cultivable bacteria varied with time (F_3,54_ = 30.5, P<0.001); it increased until day 14 and then decreased (Table S1). By contrast, the number of pseudomonads was not affected by time (F_3,54_ = 1.0, P = 0.38). The presence of amoebae did not affect the abundance of cultivable bacteria and pseudomonads (F_1,54_ = 1.2, P = 0.27 and F_1,54_ = 0.9, P = 0.36, respectively; Table S1).

The density of amoebae remained constant during the experiment, with a density of of 2–4*10^5^ amoebae per microcosm at the end of the experiment.

### Frequency of bacteria carrying biocontrol genes

**Figure 1:**
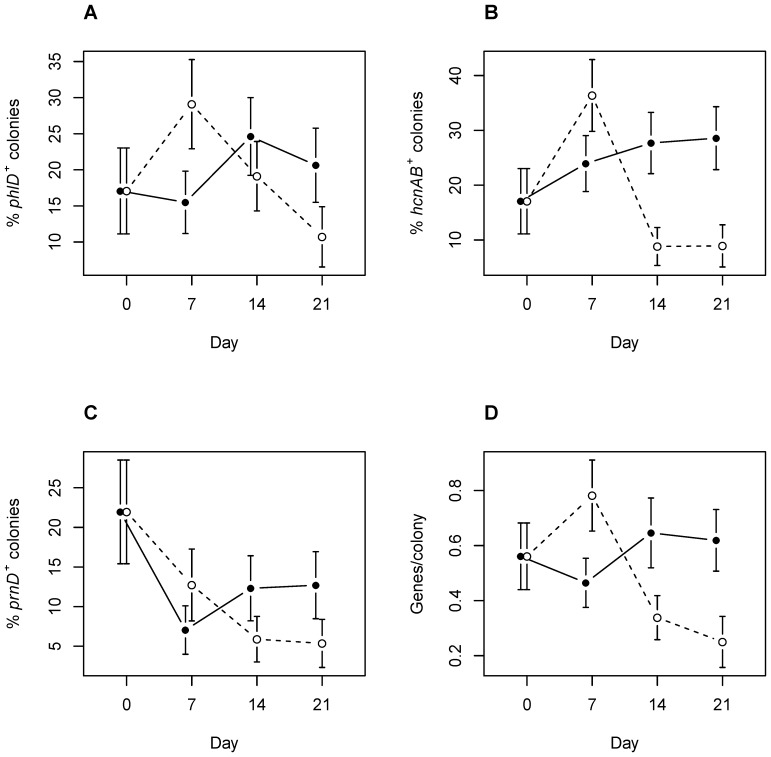
Effect of*Acanthoeba castellanii* on the frequency of *phlD*, *hcnAB* and *prnD* positive *Pseudomonas* (A–C) and on the average number of genes per bacterium (D) during the microcosm experiment with barley (means ± SE). Presence of each gene was tested by colony–PCR on isolates growing on the *Pseudomonas* specific Gould's S1 medium. closed symbols: bacterial communities co-cultivated with *Acanthamoeba castellanii*, open symbols: control treatment without protozoa.

Cultivable pseudomonads carrying the *phlD*, *hcnAB* or *prnD* genes increased in frequency over time in presence of amoebae, but tended to decline in the control treatment (Table 1, Figure 1) The proportion of bacteria harboring biocontrol genes was higher in presence of amoebae with the exception of day 7 (Table 2). This suggests that the tested biocontrol genes provided a selective advantage in presence of predators, but did not improve competitiveness against other bacteria. The effect of amoebae was more marked at later growth stages (Amoebae×Time interaction; Table 1) and varied with the tested genes. Amoebae significantly increased the frequency of bacteria carrying the *hcnAB* and *phlD* genes (Figure 1), in agreement with the importance of HCN and DAPG as antipredator defense compounds [9]. In contrast, the frequency of *prnD* positive bacteria was not significantly influenced by amoebae.

**Table 1:** Results of Poisson General Linear Models on the effects of Amoebae, Time and interaction between Amoebae and Time on the frequency of *phlD*, *hcnAB* and *prnD* positive bacteria, and on the frequency of biocontrol genes per colony in the microcosm experiment with barley.

		*phlD*positive bacteria			*hcnAB*positive bacteria			*prnD*positive bacteria		Biocontrol genes/colony		
	d.f.	F	p		F	p		F	p	F	p	
Amoebae	1	0.1	0.78		**5.4**	**0.02**	↑	0.1	0.97	2.2	0.21	
Time	3	1.8	0.61		5.0	0.18		6.0	0.12	3.8	0.43	
Amoebae×Time	2	**6.2**	**0.04**	↑	**14.0**	**0.001**	↑	4.0	0.14	**18.5**	**0.001**	↑

Significant effects are highlighted in bold (P<0.05).

↑ increase.

**Table 2:** Number of screened bacterial isolates carrying biocontrol genes in the microcosm experiment with barley.

Time (days)	Amoebae	Isolates screened	Isolates carrying biocontrol genes for			Isolates with…		
			DAPG	HCN	PRN	one	two	three
						biocontrol genes		
0	0	41	7	7	9	9	7	0
0	1	41	7	7	9	9	7	0
7	0	55	16	20	7	12	11	3
7	1	71	11	17	5	11	11	0
14	0	68	13	6	4	12	4	1
14	1	65	16	18	8	2	14	4
21	0	56	6	5	3	4	2	2
21	1	63	13	18	8	8	14	1

Amoebae also increased the average number of biocontrol genes in each isolate, and this effect increased with time (Table 1), suggesting that the ability to produce a combination of different secondary metabolites increases bacterial resistance against protozoa.

### Soil suppressiveness against*Rhizoctonia solani*

In the biocontrol assay with sugar beet we investigated if amoebae-induced shifts in biocontrol communities resulted in differences in soil suppressiveness. We followed the infection of sugar beet by *Rhizoctonia solani* in microcosms from the microcosm experiment, containing soil bacterial communities previously incubated for 21 days with or without amoebae. Plant infection by *R. solani* caused different symptoms, including brown roots, stems, leaves and snapped stems of the sugar beet germ buds (Table S2). Disease development was negatively correlated with the abundance of bacteria carrying the genes *phlD* (z = −2.445, P = 0.014) and *prnD* (z = −2.056, P = 0.039) at the beginning of the biocontrol assay, confirming the importance of these two genes for biocontrol activity. However, the presence of amoebae did not affect plant disease and even tended to increase the number of plants with brown roots (z = 0.167, P = 0.09).

## Discussion

### Effects of protozoa on bacterial communities

Predation by protozoa is a major driver of the density and functioning of bacterial communities [5], [6]. Unexpectedly, in this study amoebae did not affect the abundance of total cultivable bacteria and pseudomonads, but increased the frequency of bacteria carrying the genes responsible for the production of DAPG and HCN. These two compounds function as broad spectrum antifungal and antihelminthic metabolites, and are involved in the suppression of various root diseases [26], [27], [28], [29]. The same secondary metabolites also improve bacterial fitness in presence of protozoa [8], [9], [10]. Manipulating ecological forces favoring one function of the tested genes (bacterial fitness) can be used to obtain another, desired service provided by the same genes (inhibition of phytopathogens). Bacteria carrying more than one biocontrol gene were more abundant in presence of amoebae, suggesting that the production of multiple toxic secondary metabolites increases protection against predation. This effect of predation likely also affects the biocontrol function of the bacteria. First, bacteria producing multiple antifungal compounds are more likely to protect plants against a broader range of soil-borne pathogens than bacteria producing one single antifungal compound. Second, bacteria carrying multiple antifungal genes might be able to better persist in soil, a property that may help developing effective biocontrol inocula. The promotion of biocontrol bacteria may be further enhanced by using other microfaunal predators. The amoebae used in this study are less prey-selective than other protozoa such as flagellates [30]. More selective protozoan species may consume only non-toxic bacteria and thus promote biocontrol bacteria by eliminating competitors. Further studies are needed to identify which protozoan taxa are responsible for promoting plant beneficial soil microbial communities in field soil.

### Soil suppressiveness against*Rhizoctonia solani*

In contrast to our hypotheses, plants growing in soil previously inoculated with bacteria and amoebae developed the same levels or slightly more disease symptoms than plants growing in the control soil containing bacteria only. Nonetheless, disease development was negatively correlated with the abundance of *prnD* and *phlD* positive isolates at the end of the first microcosm experiment. This underpins the role of DAPG and PRN for inhibiting *R. solani*, but appears contradictory to the net effect of amoebae: parallel to the amoebae-mediated increase in the abundance of biocontrol bacteria we expected reduced disease development. Potentially, the lack of disease reduction resulted from the interaction between protozoa and biocontrol bacteria at early plant growth stages. In the barley microcosm experiment, protozoa reduced the establishment of biocontrol bacteria in very young seedlings, but they fostered them later. A similar effect may also have occurred in the biocontrol assay with sugar beet. Despite higher abundance of biocontrol bacteria at the beginning of the experiment, amoebae may have reduced their numbers during the early growth phase of the sugar beet seedlings, affecting plant protection. Notably, DAPG producers need to reach a threshold density of 10^5^ CFU g root^−1^ to effectively suppress pathogens [31], [32], and predation may have transiently reduced their number under this critical threshold thereby reducing protection against the pathogen in young seedlings. Further studies on the dynamics of bacteria and predators during the full plant growth cycle are needed to allow predicting plant protection by rhizosphere bacterial communities in presence of predators.

Overall, the study highlights the importance of predation as driver of the functionality of soil microbial communities with biocontrol potential. We propose that manipulating predation pressure may allow developing new strategies improving pathogen suppression and may be used to develop new biocontrol inocula with high persistence in soils.

## Supporting Information

Table S1 **Abundance of cultivable bacteria and pseudomonads at the different time points after incubation with or without** ***Acanthamoeba castellanii*** **.** Bacteria were enumerated on TSA (total heterotrophic bacteria) or Gould S1 (Pseudomonas), abundances are expressed as CFU per plants.
(DOC)

Table S2 **Number of sugar beet germ buds (out of the 8 sown in each microcosm) with infection symptoms in the biocontrol assay.**
(DOC)
