# Supplementary material for: Protozoa Drive the Dynamics of Culturable Biocontrol Bacterial Communities
Source: PLoS One. 2013 Jun 26;8(6):e66200. doi: 10.1371/journal.pone.0066200 (PMC3694078; doi:10.1371/journal.pone.0066200)
Supplement: Table S1 — Abundance of cultivable bacteria and pseudomonads at the different time points after incubation with or without Acanthamoeba castellanii . Bacteria were enumerated on TSA (total heterotrophic bacteria) or Gould S1 (Pseudomonas), abundances are expressed as CFU per plants. (DOC) [file pone.0066200.s001.doc]

Table S1: Abundance of cultivable bacteria and pseudomonads at the different time points after incubation with or without *Acanthamoeba castellanii*. Bacteria were enumerated on TSA (total heterotrophic bacteria) or Gould S1 (Pseudomonas), abundances are expressed as CFU per plants.

| **Time**  **(days)** | **Amoebae** | **CFU/plant**  **growing on TSA** | | **CFU/plant**  **growing on GOULD** | |
| --- | --- | --- | --- | --- | --- |
|  |  | **Mean** | **Standard Error** | **Mean** | **Standard Error** |
| 0 | 0 | 3.23E+07 | 2.51E+07 | 2.06E+07 | 7.66E+06 |
| 7 | 0 | 8.59E+07 | 2.51E+07 | 4.22E+06 | 7.66E+06 |
| 14 | 0 | 1.79E+08 | 2.51E+07 | 2.56E+07 | 7.66E+06 |
| 21 | 0 | 1.08E+07 | 2.68E+07 | 3.77E+06 | 8.19E+06 |
| 0 | 1 | 3.23E+07 | 2.51E+07 | 2.06E+07 | 7.66E+06 |
| 7 | 1 | 7.75E+07 | 2.51E+07 | 6.46E+06 | 7.66E+06 |
| 14 | 1 | 1.39E+08 | 2.51E+07 | 9.38E+06 | 7.66E+06 |
| 21 | 1 | 1.68E+07 | 2.68E+07 | 6.64E+06 | 8.19E+06 |
