# Supplementary material for: Protozoa Drive the Dynamics of Culturable Biocontrol Bacterial Communities
Source: PLoS One. 2013 Jun 26;8(6):e66200. doi: 10.1371/journal.pone.0066200 (PMC3694078; doi:10.1371/journal.pone.0066200)
Supplement: Table S2 — Number of sugar beet germ buds (out of the 8 sown in each microcosm) with infection symptoms in the biocontrol assay. (DOC) [file pone.0066200.s002.doc]

Table S2: Number of sugar beet germ buds (out of the 8 sown in each microcosm) with infection symptoms in the biocontrol assay.

| **Time** | **Amoebae** | **Germ buds with …** | | | | | | | | **Completely brown** | |
| --- | --- | --- | --- | --- | --- | --- | --- | --- | --- | --- | --- |
| **(days)** |  | **brown roots** | | **brown stems** | | **brown leaves** | | **snapped stems** | | **germ buds** | |
|  |  | **Mean** | **SE** | **Mean** | **SE** | **Mean** | **SE** | **Mean** | **SE** | **Mean** | **SE** |
| 6 | 0 | 1.250 | 0.514 | 0.000 | 1.052 | 0.000 | 0.927 | 0.000 | 0.758 | 0.000 | 0.750 |
| 7 | 0 | 2.000 | 0.514 | 0.125 | 1.052 | 0.000 | 0.927 | 0.000 | 0.758 | 0.000 | 0.750 |
| 8 | 0 | 3.250 | 0.514 | 0.625 | 1.052 | 0.125 | 0.927 | 0.000 | 0.758 | 0.000 | 0.750 |
| 9 | 0 | 4.750 | 0.514 | 1.000 | 1.052 | 0.375 | 0.927 | 0.000 | 0.758 | 0.000 | 0.750 |
| 10 | 0 | 4.750 | 0.514 | 1.000 | 1.052 | 0.375 | 0.927 | 0.000 | 0.758 | 0.000 | 0.750 |
| 11 | 0 | 4.750 | 0.514 | 1.000 | 1.052 | 0.375 | 0.927 | 0.000 | 0.758 | 0.000 | 0.750 |
| 12 | 0 | 6.500 | 0.514 | 2.625 | 1.052 | 1.875 | 0.927 | 1.125 | 0.758 | 0.500 | 0.750 |
| 13 | 0 | 7.250 | 0.514 | 2.625 | 1.052 | 1.875 | 0.927 | 1.625 | 0.758 | 0.875 | 0.750 |
| 14 | 0 | 7.500 | 0.514 | 2.625 | 1.052 | 2.875 | 0.927 | 1.625 | 0.758 | 1.000 | 0.750 |
| 15 | 0 | 7.500 | 0.514 | 2.625 | 1.052 | 3.250 | 0.927 | 1.875 | 0.758 | 1.250 | 0.750 |
| 16 | 0 | 7.500 | 0.514 | 2.875 | 1.052 | 3.625 | 0.927 | 1.875 | 0.758 | 1.250 | 0.750 |
| 17 | 0 | 7.500 | 0.514 | 2.875 | 1.052 | 3.625 | 0.927 | 1.875 | 0.758 | 1.250 | 0.750 |
| 18 | 0 | 7.500 | 0.514 | 2.875 | 1.052 | 3.625 | 0.927 | 1.875 | 0.758 | 1.250 | 0.750 |
| 19 | 0 | 7.500 | 0.514 | 3.000 | 1.052 | 3.875 | 0.927 | 2.125 | 0.758 | 2.500 | 0.750 |
| 6 | 1 | 2.125 | 0.514 | 0.000 | 1.052 | 0.000 | 0.927 | 0.000 | 0.758 | 0.000 | 0.750 |
| 7 | 1 | 3.375 | 0.514 | 0.000 | 1.052 | 0.000 | 0.927 | 0.000 | 0.758 | 0.000 | 0.750 |
| 8 | 1 | 3.875 | 0.514 | 0.250 | 1.052 | 0.000 | 0.927 | 0.000 | 0.758 | 0.000 | 0.750 |
| 9 | 1 | 5.625 | 0.514 | 0.750 | 1.052 | 0.000 | 0.927 | 0.000 | 0.758 | 0.000 | 0.750 |
| 10 | 1 | 5.625 | 0.514 | 0.750 | 1.052 | 0.000 | 0.927 | 0.000 | 0.758 | 0.000 | 0.750 |
| 11 | 1 | 5.625 | 0.514 | 0.750 | 1.052 | 0.000 | 0.927 | 0.000 | 0.758 | 0.000 | 0.750 |
| 12 | 1 | 7.875 | 0.514 | 2.000 | 1.052 | 1.625 | 0.927 | 0.250 | 0.758 | 1.000 | 0.750 |
| 13 | 1 | 8.000 | 0.514 | 3.375 | 1.052 | 3.000 | 0.927 | 2.250 | 0.758 | 2.250 | 0.750 |
| 14 | 1 | 8.000 | 0.514 | 3.375 | 1.052 | 3.625 | 0.927 | 2.250 | 0.758 | 2.375 | 0.750 |
| 15 | 1 | 8.000 | 0.514 | 4.000 | 1.052 | 3.875 | 0.927 | 2.750 | 0.758 | 2.625 | 0.750 |
| 16 | 1 | 8.000 | 0.514 | 4.125 | 1.052 | 4.125 | 0.927 | 2.750 | 0.758 | 2.625 | 0.750 |
| 17 | 1 | 8.000 | 0.514 | 4.125 | 1.052 | 4.125 | 0.927 | 2.750 | 0.758 | 2.625 | 0.750 |
| 18 | 1 | 8.000 | 0.514 | 4.125 | 1.052 | 4.125 | 0.927 | 2.750 | 0.758 | 2.625 | 0.750 |
| 19 | 1 | 8.000 | 0.514 | 4.375 | 1.052 | 4.500 | 0.927 | 3.250 | 0.758 | 3.125 | 0.750 |
